# Zellweger spectrum disorders: clinical manifestations in patients surviving into adulthood

**DOI:** 10.1007/s10545-015-9880-2

**Published:** 2015-08-19

**Authors:** Kevin Berendse, Marc Engelen, Sacha Ferdinandusse, Charles B. L. M. Majoie, Hans R. Waterham, Frédéric M. Vaz, Johannes H. T. M. Koelman, Peter G. Barth, Ronald J. A. Wanders, Bwee Tien Poll-The

**Affiliations:** Department of Paediatric Neurology, Emma Children’s Hospital, Academic Medical Centre (AMC), University of Amsterdam, Meibergdreef 9, 1105 AZ Amsterdam, The Netherlands; Laboratory Genetic Metabolic Diseases, Emma Children’s Hospital, AMC, University of Amsterdam, Amsterdam, The Netherlands; Department of Radiology, AMC, University of Amsterdam, Amsterdam, The Netherlands; Department of Neurology and Clinical Neurophysiology, AMC, University of Amsterdam, Amsterdam, The Netherlands

## Abstract

**Introduction:**

We describe the natural history of patients with a Zellweger spectrum disorder (ZSD) surviving into adulthood.

**Methods:**

Retrospective cohort study in patients with a genetically confirmed ZSD.

**Results:**

All patients (*n* = 19; aged 16–35 years) had a follow-up period of 1–24.4 years (mean 16 years). Seven patients had a progressive disease course, while 12 remained clinically stable during follow-up. Disease progression usually manifests in adolescence as a gait disorder, caused by central and/or peripheral nervous system involvement. Nine were capable of living a partly independent life with supported employment. Systematic MRI review revealed T2 hyperintense white matter abnormalities in the hilus of the dentate nucleus and/or peridentate region in nine out of 16 patients. Biochemical analyses in blood showed abnormal peroxisomal biomarkers in all patients in infancy and childhood, whereas in adolescence/adulthood we observed normalization of some metabolites.

**Conclusions:**

The patients described here represent a distinct subgroup within the ZSDs who survive into adulthood. Most remain stable over many years. Disease progression may occur and is mainly due to cerebral and cerebellar white matter abnormalities, and peripheral neuropathy.

## Introduction

Zellweger spectrum disorders (ZSDs, OMIM #601539) constitute a subgroup of the peroxisome biogenesis disorders and represent a clinical continuum from severe to relatively milder phenotypes. ZSDs are caused by pathogenic mutations in at least 13 different *PEX* genes, encoding peroxins. Generally, there is genotype-phenotype correlation (Rosewich et al 2005) (Moser 1999). Patients with the severe phenotype present in the neonatal period with failure to thrive, jaundice, hypotonia and dysmorphic features (Weller et al 2003) and high mortality within the first year of life. This severe phenotype was described as Zellweger syndrome (Bowen et al 1964). Patients with a milder phenotype display a more variable symptomatology and age of presentation, but the onset is usually in early childhood. The most common presenting signs are developmental delay, with visual loss from retinal degeneration, sensorineural hearing loss and liver disease (Baumgartner et al 1998). The majority of ZSD patients with a mild phenotype have normal MRI findings during childhood, but ultimately develop leukoencephalopathy later in life (Poll-The and Gärtner 2012).

Definitive diagnosis requires laboratory investigations to assess peroxisomal functions, and confirmation by enzymatic analysis in fibroblasts, and/or PEX gene mutation studies.

Only limited data are available on the phenotype and natural history in adults with ZSDs (Rosewich et al 2005) (Mignarri et al 2012) (Matsui et al 2013) (Raas-Rothschild et al 2002) (Régal et al 2010). To improve the accuracy of clinical diagnosis and to enable comparison of future evaluations of therapeutic interventions, we describe the natural history of a cohort of 19 patients (≥16 years) with molecularly defined ZSDs.

## Material and methods

### Patients

Retrospective clinical and laboratory data were collected from 19 ZSD patients. The diagnosis was confirmed by biochemical and molecular testing. Clinical and biochemical data were collected at many time points between 1991 and 2014. All patients were seen in the Academic Medical Centre in Amsterdam and examined by the authors (ME, PGB, or BTPT). Some data from patients 1, 3–6, 8, 13–14, 16–18 (Poll-The et al 2004) and patient 4 (Ebberink et al 2012) were reported previously. Age at diagnosis was defined as biochemically confirmed diagnosis, and not the onset of symptoms.

### Hearing and visual assessment

Objective methods (brainstem audiometry evoked response and visually evoked potentials) and subjective methods (free-field audiometry and Snellen chart, with 1.0 scored as normal vision (Peters 1961)) were used to asses hearing and visual impairment.

### Imaging

A total of 39 MRI studies of the brain were performed in 16 patients. The studies were performed on a 1.5 T MR scanner using the following sequences: axial, sagittal and coronal spin-echo T1-weighted, T2-weighted and axial fluid-attenuated inversion recovery image. The first MRI scans of patients 8, 9, and 16–19, the first and second MRI’s of patients 1, 3, and 5 and the fourth MRI of patient 6 were described earlier (Barth et al 2004). Liver cirrhosis was diagnosed using a Fibroscan, as described previously (Foucher et al 2006).

### Laboratory tests

To establish whether a patient is affected by a ZSD several peroxisomal metabolites, reflecting the in vivo function of peroxisomes, were determined in blood, including levels of VLCFA, phytanic and pristanic acid (Vreken et al 1998), pipecolic acid (Rashed et al 2001) and bile acids (Bootsma et al 1999). In erythrocytes, plasmalogen (C16:0- and C18:0-dimethyl acetal) levels were measured (Dacremont and Vincent 1995) and levels of oxalic acid and glycolic acids were determined in urine spots and corrected for creatinine excretion (Wolthers and Hayer 1982). A total of 332 biochemical analyses were performed in plasma, ranging from two (patient 4) to 43 (patient 18) tests per patient. Adrenal insufficiency was diagnosed by means of an adrenocorticotropic hormone stimulation (Synacthen) test (Berendse et al 2014).

Several peroxisomal parameters were studied in cultured skin fibroblasts including, catalase immunofluorescence microscopy (Wanders et al 1989), VLCFA profile (Dacremont et al 1995), peroxisomal alpha- and beta-oxidation activity (Wanders et al 1995a) and dihydroxyacetonephosphate-acyltransferase (DHAPAT) activity (Wanders et al 1995b).

### Mutation analysis

Complementation analysis in cultured skin fibroblasts was used to determine the defective *PEX* gene, followed by Sanger sequencing on genomic DNA (Ebberink et al 2011).

### Statistical analysis

Mann–Whitney U tests were performed using the IBM Statistical package for the Social Sciences (SPSS) software version 20 (IBM, U.S.A.).

### Standard protocol approvals, registrations and patient consents

For this project the IRB issued a waiver since the study is retrospective and only anonymized data was used. Written informed consent was obtained from the parents of all the patients, and authorization for disclosure of recognizable persons in photographs for publication were obtained.

## Results

The detailed clinical characteristics of the 19 patients from 16 families are summarized in Tables 1 and 2. All patients, nine females and ten males, were of Caucasian background and no parental consanguinity was reported. To obtain a framework for comparing the severity of the neurologic disorder with the MRI findings and biochemical abnormalities we delineated two broad categories on the basis of the degree of communication. Patients in group 1 (patients 1–12) were able to communicate with structured grammatical speech and patients in group 2 (patients 13–19) had no structured speech. Age at diagnosis varied between 7 days and 31 years (mean group 1: 7.6 years, group 2: 1.2 years). The diagnosis of patients 3 and 16 was at an early age because they had an affected older sibling. In two other patients (2 and 4) there was a significant delay before diagnosis, due to normal peroxisomal metabolites in plasma and a clinical presentation atypical for a ZSD (Ebberink et al 2012).

**Table 1:** Clinical and genetic characteristics of 19 ZSD patients (group 1: patients 1–12; group 2: patients 13–19)

	No. 1(A)	No. 2	No. 3(A)	No. 4	No. 5	No. 6	No. 7	No. 8(B)	No. 9(B)	No. 10	No. 11	No. 12	No. 13(C)	No. 14	No. 15	No. 16(C)	No. 17	No. 18^a^	No. 19
Mutations																			
Allele 1	P1c.2528G>	P1c.2528G>A	P1c.2528G>A	P11Bc.64C>T	P26c.292C>T	P1c.2528G>A	P1c.1777G>A	P1c.2528G>A	P1c.2528G>A	P6c.1801C>T	P1c.2528G>A	P26c.292C>T	P1c.2528G>A	P1c.2528G>A	P1c.2528G>A	P1c.2528G>A	P1c.2528G>A	P1c.2528G>A	P1c.2528G>A
Allele 2	P1c.2528G>	P1c.2528G>A	P1c.2528G>A	P11Bc.64C>T	P26c.292C>T	P1c.2528G>A	P1c.2071+1G>T	P1c.2528G>A	P1c.2528G>A	P6c.1992G>C	P1c.2528G>A	P26c.292C>T	unknown	P1c.2528G>A	P1c.2528G>A	unknown	P1c.2636 T>C	P1c.2097insT	P1c.2528G>A
Age at latest visit (yrs)	35	32	31	27.5	23	22	19.5	19	17.5	17.5	17	16	28	27	24.5	22	18.5	18	17
Age at diagnosis (yrs)	5	31	0.25	25	6	2	0.25	3.5	3	3	8	4	0.5	0.75	0.02	0.02	1.3	0.5	5
Presenting symptoms																			
Failure to thrive													+	+	+	+		+	
Bilateral 4-fingerline								+				+		+					
Hearing impairment	+	+						+	+	+	+	+							+
Hypotonia						+								+	+			+	
Jaundice			+		+	+							+	+		+			+
Visual impairment	+	+	+	+	+		+			+	+	+		+	+			+	
Facial dysmorphia																			
Attached earlobes	+	+	+		+	+			+			+			+	+	+	+	+
Epicanthal folds											+							+	+
High forehead	+		+					+				+		+					+
Speech development																			
Absent														+	+	+	+	+	+
Short sentences		+			+								+						
Sufficient working vocabulary	+		+	+		+	+	+	+	+	+	+							
Intellectual disability	moderate	moderate	moderate	moderate	moderate	moderate	moderate	moderate	moderate	moderate	none	moderate	severe	severe	severe	severe	severe	severe	severe
Age of examination in yrs	7	32	18		11	15	5	6	14		9	15		9			15		9
Total IQ						54	67		64			60							
Verbal IQ			<55		54			67			105								
Non-verbal IQ			60		54			59			83								
Developmental level in yrs	2.5	7												2.5			1		2
Residence																			
Assisted living facility	+	+	+																
Parental home				+		+	+	+	+	+	+	+							
Parental home with continuous supervision					+								+	+		+			+
nursing home															+		+	+	
Education																			
Higher vocational education											+								
Sec. vocational education level 1^b^								+	+										
Special education cluster 2^c^	+		+			+	+			+		+							
Special education cluster 3^d^		+		+	+														
Supported employment ^e^	+	+	+	+		+		+				+							
Miscellaneous	EH	EH	EH	EH	EH	EH, explicit memory at a normal level	EH	EH, able to cope with money, drives a scooter	EH, drives a bicycle	EH	EH, completely normal gait	EH, able to cope with money	EH	EH, examined during house call	EH	EH, PEG tube (at 7 years of age)	EH	EH, PEG tube (at 5 years of age)	EH

*Abbreviation: EH* enamel hypoplasia, *P* PEX, *IQ* intelligence quotient, *PEG* percutaneous endoscopic gastrostomy

(A) sibs; (B) sibs; (C) sibs

^a^Patient died at 18 years

^b^Education for specific trades or occupations, ascending difficulty of access criteria from level 1 to 4

^c^School for deaf and partially hearing children, or children with serious speech and language difficulties

^d^School for children with serious learning difficulties, long-term condition and somatic problems, physical handicap and a physical handicap

^e^Paid work for mentally or physically disabled persons, taking place in regular or normal work settings, with continuous supervision

**Table 2:** Clinical characteristics of 19 ZSD patients (group 1: patients 1–12; group 2: patients 13–19)

Nervous system	No. 1 (A)	No. 2	No. 3 (A)	No. 4	No. 5	No. 6	No. 7	No. 8 (B)	No. 9 (B)	No. 10	No. 11	No. 12	No.13(C)	No. 14	No. 15	No. 16 (C)	No. 17	No. 18^a^	no. 19
Visual system																			
Cataract				+	+														
Retinitis pigmentosa	+	+	+		+	+	+	+	+	+	+	+		+		+	+	+	+
Visual acuity scales^b^	0.33	0.05	0.08	0.1	0.11	0.1	0.1	0.2	0.25	0.25	0.25	0.15	0.2	0.05	0.04	<0.1	0.1	0.05	
Stable/progressive	=	↓	↓		↓	=	=	=	=	=	=	=	=	=	=	=	=	↓	=
VEP, age at examination in yrs	30		26			18	2	14	13	4	11	11			1		5	1	
Flash	normal		PL			PL	normal	PL		DL	PL	PL					PL	poor pattern	
Pattern	PL		PL					PL	DL	DL							PL		
Hearing system										CI									
Hearing loss (dB),left/right		80			90/110		80/75		80		85	70		90/90	80		95		impossible to asses
Low frequency	60		80					75		40								80	
High frequency	110		80					75		110								80	
Stable/progressive	=	=	=		↓	↓	=	=	=	=	=	=	↓	=	=		=		=
BAEP, age at examination in yrs	30		26			18		14	13		11	11		2	1			2	
Absent	+		+			+		+	+		+	+		+					
Normal															+			+	
Motor and sensory system		CS, PS		PN	CS, PN, PS	CS, PS	CS		CS, PN	CS, PN		CS	PN		PS	CS, PN	PS	CS, PN, PS	
Ataxia		+				+	+		+	+		+						+	
Babinski					+	+									+		+	+	
Brisk reflexes		+				+	+				+				+			+	
Clonus						+									+				
Dysarthria		+			+	+			+										
Dysmetria					+	+						+						+	
Intention tremor					+		+												
Nystagmus		+				+	+			+		+					+	+	+
Absent reflexes				+						+									
Clawfeet				+															
Contractures ankles/knee															+	+			
Hammertoes		+		+						+			+						
Muscular atrophy				+	+				+				+	+		+		+	
Pes cavus		+		+					+	+			+			+	+		
Other signs				absent vibration sense					steppage gait							unable to walk		no reaction upon pain stimuli	
NCS, age at examination in yrs	30				16	18				14		11						4	
Normal	+					+						+						+	
Sensory responses																			
Demyelinating PN					+					+									
Other systemic features													LC			LC	LF	AI, HM, KS, LC, SM	
Maximal motor skills																			
Wheelchair-bound					+								+		+	+	+	+	
Sitting					+											+		+	
Walking with support													+		+				
Walking		+				+											+		
Running	+		+	+			+	+	+	+	+	+		+					+
Disease course in recent years	=	=	=	↓	↓	↓	=	=	↓	=	=	=	=	=	=	↓↓	↓	↓↓	=
Clinical deterioration/age in yrs				PN/26	ataxia/15	ataxia/16			in MF/16							in MF/12	in MF/16	in MF/14	

*Abbreviation: AI* adrenal Insufficiency, *BAEP* brainstem acoustic evoked potentials, *CS* cerebellar syndrome, *DL* delayed latency, *HM* hepatomegaly, *KS* kidney stones, *LC* liver cirrhosis, *LF* liver fibrosis, *MF* motor function, *NCS* nerve conduction studies, *PL* prolonged latency, *PS* pyramidal signs, *PN* polyneuropathy, *VEP* visually evoked potentials, *SM* splenomegaly

“=” = stable; ↓ = slowly progressive; ↓↓ = progressive

(A) sibs; (B) sibs; (C) sibs

^a^Patient died at 18 years

^b^Tests are expressed as Snellen equivalent

We obtained information about clinical features, biochemical profile, education, current level of social functioning and help required for activities of daily living.

### Clinical features

During infancy, seven of the 19 patients had prolonged neonatal jaundice, four presented with hypotonia, five with failure to thrive, 12 had a visual handicap due to retinal degeneration and eight patients presented with hearing impairment (Table 1). During childhood, all patients had a moderate to severe developmental delay as well as visual and hearing loss. Current ages ranged from 16 to 35 years (mean 22.5 years). Patient 18 died from liver failure at 18 years. At last follow-up, all patients had nyctalopia and retinopathy, caused by retinitis pigmentosa in 16/19. Visual acuity ranged from 4 to 33 % (mean 14 %) with 100 % being normal vision and 5 % defined as legally blind in Europe (Kocur and Resnikoff 2002). The visual impairment was progressive in four and stable in 14 patients. All had gross sensorineural hearing deficits, ranging from a loss of 60 dB to 110 dB and 17 patients (except patients 15 and 18) used hearing aids with clinical benefit. Ambulation ranged from running (11/19) to walking unsupported (3/19), walking with support (2/19) and the ability to sit unsupported (3/19). Patient 5 had normal motor function during childhood. However, since the age of 15 years he lost the ability to walk independently due to progressive cerebellar ataxia and weakness caused by peripheral neuropathy. At the age of 21 he developed pyramidal signs and became completely wheelchair-bound at 23 years. Patient 18 was able to walk independently at 4 years of age, but at the age of 14 years she developed progressive cerebellar and sensory ataxia and lost independent ambulation.

Specific findings included cerebellar signs in 9/19, pyramidal signs in 5/19 and signs of peripheral neuropathy in 11/19 patients (Table 2). The peripheral neuropathy could only be confirmed by nerve conduction studies in two patients (5 and 10), due to reluctance of the parents to allow latter studies. In these two patients the neuropathy was classified as a demyelinating neuropathy, according to established criteria with a motor conduction velocity below 41 ms in the median nerve and 35 ms in the peroneal nerve (Van Asseldonk et al 2005). Overall, seven had progressive neurological symptoms (mainly worsening of the gait disorder) and 12 were stable at most recent follow-up. Epilepsy was not seen in any of the patients.

Non-neurological findings included splenomegaly, hepatomegaly, renal pelvic stones and adrenal insufficiency in one patient and liver cirrhosis in 3/19. All patients had enamel hypoplasia.

### Activities of daily living

Of the 19 patients, six lived in assisted-care facilities (Table 1), of whom patients 15 and 17–18 lived in a round the clock care facility and were completely dependent on others (i.e., nursing home). The remaining three were self-supporting with respect to daily life activities, albeit under supervision. The other 13 patients lived with their parents, with or without continuous supervision. Of these patients, only patients 13 and 14 were not able to feed themselves independently. All patients except for patient 11 had a minor to severe gait disorder, limiting their physical mobility. Cognition varied widely from normal cognitive abilities in patient 11, to moderate or severe intellectual disability in most.

### MRI findings

**Fig. 1:**
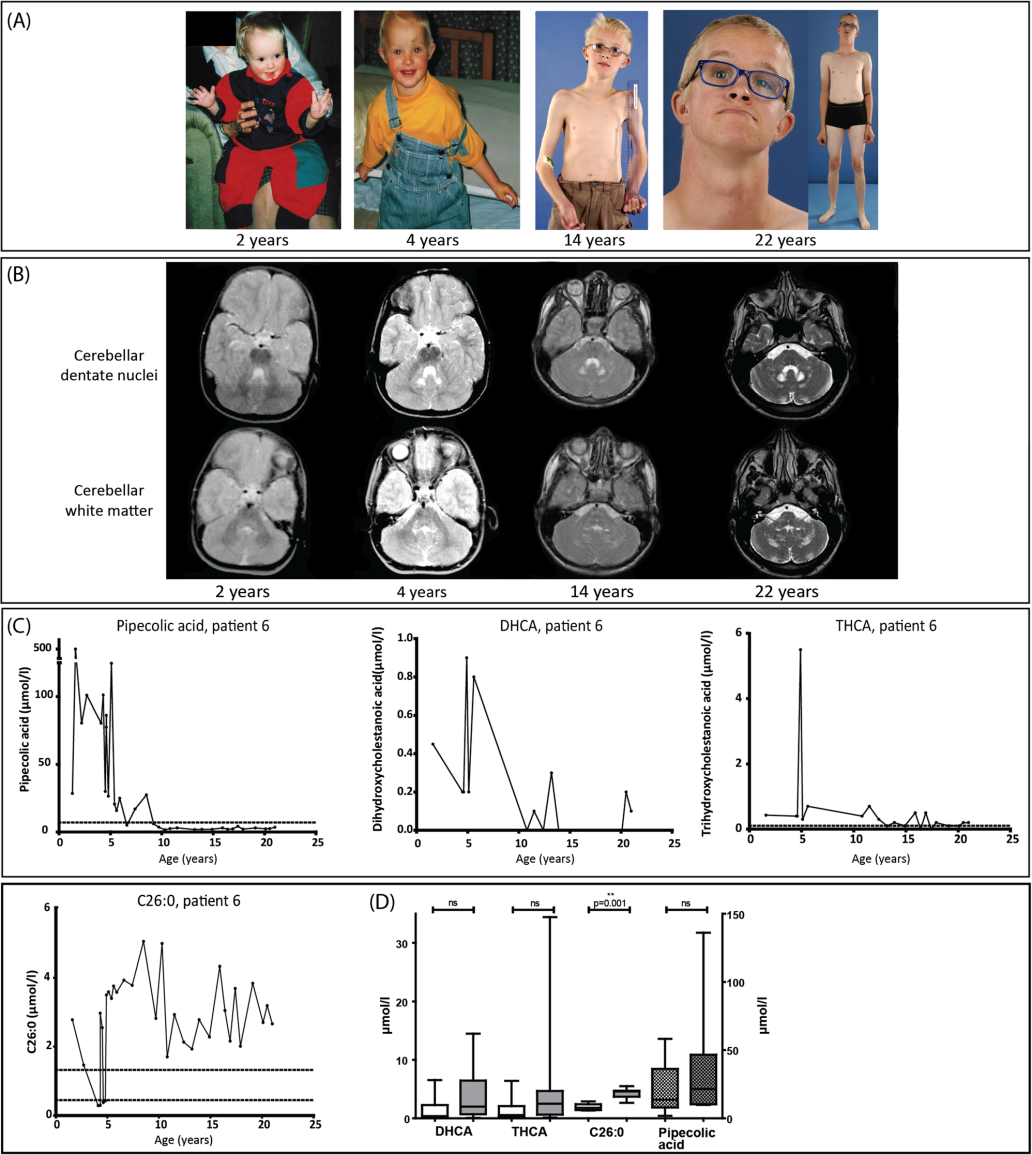
**a**–**c** Growth, development, brain MR images and biochemical parameters of patient 6. **a** At the age of 2 and 4 years patient 6 was completely normal and only presented a mild developmental delay and visual impairment. Since the age of 16 years, a decline in disease progression was noted and this patient developed progressive pyramidal tract symptoms with a gait ataxia. **b** Leukoencephalopathy with slowly progressive disease course. Axial T2-weighted MR images at level of cerebellar dentate nuclei and cerebellar white matter show bilateral progressive white matter hyperintensities of the hilus of the dentate nucleus and central white matter of both hemispheres between 4 and 22 years old. MR images at 2 years old were normal. **c** Fluctuations of the biochemical parameters measured in plasma at different time points, which are representative for the other patients. Note that both DHCA and THCA were normal at the age of 15 years but abnormal at 21 years. The reference range of pipecolic acid is 0.1–7 μmol/l, of THCA 0.0–0.1 μmol/l and of C26:0 0.45–1.32 μmol/l. The levels of DHCA are not detectable in controls. **d** Boxplot showing median, interquartile range, minimal and maximal range of the average level of DHCA, THCA, C26:0 (all *left axis*) and pipecolic acid (*right axis*) in plasma per patient throughout life between group 1 (*white* and *white dotted bars*) and 2 (*gray* and *gray dotted bars*). A total of 332 biochemical analyses were performed, ranging from 2 (patient 4) to 43 (patient 18) tests per patient. Statistical analyses were performed with a Mann–Whitney *U* test. *Abbreviation: ns*, not significant

Cerebral MRI scans were available from 16 patients, with follow-up in 10 (Table 3). T2-weighted MR images showed progressive hyperintense white matter changes in the cerebral hemispheres in 4/16. These were located around the supratentorial ventricular system merging with surrounding normal white matter without sharp demarcation. Patient 6 (Fig. 1) had additional lesions in the corpus callosum and in the posterior limbs of the internal capsules. Follow-up images showed progressive central cerebellar white matter changes on T2-weighted images between 11 and 22 years. Furthermore, 8/16 patients had T2 hyperintensities in the areas surrounding the cerebellar dentate nuclei, which was progressive in 3 (patients 3, 5–6). T2-hyperintensity in the cerebellar white matter was seen in 6 patients, and was progressive in one (patient 6). Two patients (16, 18) had supratentorial ventricular dilatation and 6/16 presented with cerebellar cortical atrophy, of whom 3 patients (3, 16, 18) showed concomitant supratentorial cortical atrophy.

**Table 3:** Brain magnetic resonance imaging characteristics in 19 ZSD patients

Patients no.	Age at MRI, yrs	White matter hyperintensity^a^			Cerebellar cortical atrophy
		Cerebral hemispheres	Cerebellum		
			HDN	WM	
1	14	─	─	─	─
	21	─	─	─	─
	30	─	─	─	─
2	32	─	+ +	+ +	─
3	10	─	─	─	─
	16	+	+	+	─
	25	+ +	+ +	+	+
4	12	─	─	─	─
5	7	─	+	+	+
	13	─	+	+	+
	21	─	++	+	++
6	0.5	─	─	─	─
	2	─	─	─	─
	4	─	+	─	─
	11	+ +	+ +	─	─
	14	+++	+++	+	─
	17	++++	++++	++	─
	18	+++++	+++++	+++	─
	22	+++++	+++++	++++	─
7	14	─	─	─	─
8	4	─	─	─	─
	13	─	+	─	+
	17	─	+	─	+
9	3	─	─	─	─
	6	─	─	─	─
	13	++	++	++	─
	16	++	++	++	─
10	none				
11	3	─	─	─	─
	15	─	─	─	─
12	6	─	+	─	─
	11	─	+	─	─
	16	─	+	─	─
13	none				
14	none				
15	1	─	─	─	─
16	7	─	─	─	+
17	7	+	+	─	─
18	1	─	─	─	─
	17	─	─	+	+
19	3	─	─	─	─
	9	─	─	─	+

*Abbreviation: HDN* hilus of the dentate nucleus, *WM* cerebellar white matter

“−” = absent; “+” = The number of plus signs indicates disease severity

^a^T2-weighted images

### Laboratory tests

Detailed individual biochemical profiles, during the time of diagnosis and last follow-up, are described in Tables 4 and 5. At the last available measurement the most important abnormalities in plasma included elevated levels of C26:0 (16/19 patients), pipecolic acid (10/19 patients), DHCA (9/19 patients) and THCA (6/19 patients). There were two patients (patients 2 and 11) with only one mildly abnormal peroxisomal biomarker in plasma and erythrocytes. Compared with the biochemical results at time of diagnosis, we observed a decline in the levels of DHCA and THCA (15/19 patients), C29-dicarboxylic acid (13/14 patients) and pipecolic acid (10/19 patients), and in some patients even a complete normalization (patients 6–11, 14, 19).

**Table 4:** Biochemical Analyses in plasma, erythrocytes and urine from ZSD patients

		Patient 1 (A)		Patient 2		Patient 3 (A)		Patient 4		Patient 5		Patient 6		Patient 7		Patient 8 (B)		Patient 9 (B)		Patient 10		Patient 11		Patient 12		Patient 13 (C)		Patient 14		Patient 15		Patient 16 (C)		Patient 17		Patient 18		Patient 19	
Age, yrs	Ref. range	12^a^	35^b^	31^a^	33^b^	8^a^	31^b^	26^a^	27.5^b^	8^a^	23^b^	1.6^a^	23^b^	4^a^	19.5^b^	4^a^	19^b^	4^a^	17.5^b^	3.5^a^	17.5^b^	8^a^	17^b^	5.5^a^	16^b^	11.5^a^	28^b^	8.5^a^	18^b^	birth^a^	24^b^	1^a^	22^b^	1.5^a^	16^b^	0.5^a^	18^b^	5^a^	17^b^
Plasma																																							
C22:0, μmol/l	40-119	**14**	26.2	51	72.5	**9.6**	**38.2**	65	53.34	**36**	**27.4**	**12.1**	77.1	**18**	**34.59**		54.5	**31.6**	42.78	**17.2**	40.18	46.8	61.7	**16**	**20.3**	45.8	56.4	**19.7**	**38**	**19**	94.2	**10.5**	**31.6**	**15**	**32.2**	76	**22.7**	**29**	49.6
C24:0, μmol/l	33-84		37	**45**	57.3		**44.3**	75.6	48.8	37	**29.5**	**17.1**	78.8	**21**	39.93	34	57.9	40.2	48.31	**18.2**	37.84	47.4	58.9	**14**	**25.2**	44.5	65.5	45	39	**32**	84.1	**19.7**	**29.3**	**22**	34.5		33.9	39	54.8
C26:0, μmol/l	0.45–1.32	0.5	**3.31**	**2.2**	0.73	0.5	1.16	1.3	**1.38**	**4**	**2.73**	**2.78**	**3.51**	**1.5**	**1.67**	**1.8**	**1.63**	**3.01**	**2.35**	**1.63**	**1.33**	**1.91**	1.28	**1.47**	**1.43**	**2.64**	**4.72**	**1.38**	**1.9**	**2.8**	**2.44**	**3.15**	**2.4**	**5.6**	**3.16**	0.89	**4.63**	**3.3**	**4.17**
C26:0/C22:0, ratio	0–0.02	**0.04**	**0.13**	**0.04**	0.01	**0.05**	**0.05**	0.02	**0.03**	**0.11**	**0.1**	**0.23**	**0.05**	**0.08**	**0.05**		**0.03**	**0.1**	**0.05**	**0.1**	**0.03**	**0.04**	0.02	**0.09**	**0.07**	**0.06**	**0.08**	**0.07**	**0.05**	**0.15**	**0.03**	**0.3**	**0.08**	**0.36**	**0.1**	0.01	**0.2**	**0.11**	**0.08**
CA, μmol/l	0.1–4.7		0.8	0.8	0.6		0.1		1.7	2.6	1	**10.7**	0.5	1.5	0.1	2.9	1	0.8	2	1.2	0.4	**6.7**	0.3	1.9	1.4	2.6	0.8	1.7	1.4	3.6	1.5	**10.9**	1.4	2.5	1.5	**32**	**48.2**	**6.3**	0.6
CDCA, μmol/l	0.7–10		2.6	3.6	2		0.6		0.6	0.2	0.3	4.2	1.3	**12**	0.3	0.4	5.4	3	**11.5**	4.1	1.5	3.3	0.6	10	4.8	0.4	0.2	0.7	0.6	2.8	0.6	3.3	1.5	8.4	6.8	5.5	8.2	**14**	0.9
DHCA, μmol/l	0	**0.2**	**1.6**	0	0	**0.2**	**0.3**	0	0	**10**	**3.1**	**0.5**	0	**6.1**	0	**1.1**	0	**0.6**	0	**0.9**	0	**0.4**	0	**22.4**	**2.8**	**2.3**	**0.1**	**0.1**	0	**0.3**	**0.1**	**2.9**	**1.2**	**6**	**2.2**	**47**	**4.5**	**1.2**	0
THCA, μmol/l	0–0.1	**0.2**	**0.9**	0	0	**0.3**	0.1	0	0	**1.9**	**1.2**	**0.4**	0	**6.2**	0	**0.6**	0.1	**0.3**	0.1	**1.7**	0.1	**1.9**	0	**15.1**	**7.2**	**1.8**	0.1	0.1	0.1	**10.3**	**0.1**	**9.5**	**0.2**	**3.5**	**1.1**	**334**	**7.4**	**2.2**	0
C29, μmol/l	0		**0.1**		0		0	0	0	**1.7**	**0.2**	**0.3**	0	**1.2**	0	**1.2**	0	**0.1**	0	**0.1**	0	**0.1**	0	**0.4**	**0.1**	**0.9**	0		0		0	**4.4**	0	**2.3**	**0.1**	**25**	**0.3**	**0.3**	0
Pipecolic acid, μmol/l	0.5–9.9		**10.8**		**20.4**		3.5	1.4	1.8		**38.6**	**500**	2.9	**111**	3		3.7	**13.7**	7.6	**33.6**	**10.2**	**10.5**	5.5	**36.1**	**46.1**	**15.8**	**32**	**21.4**	3.3	**14.1**	2.9		**14.7**	**283**	**10.7**	**230**	**53.3**	**27**	**13**
Pristanic acid, μmol/l	0.1–3	2.6	**4.3**	2.9	0.2	**5.5**	0.9	0.6	0.5	**26**	**7.7**		0.8	2	0.3	**21**	0.6	**3.2**	1.5	0	1.1	**16.6**	0.1	**16.7**	**15.9**	**6**	**5.4**		0.2	0.4	2		0.8	**44**	2.9	2.6	0.4	**9.7**	**7.9**
Phytanic acid, μmol/l	0.1–7	**4.4**	**10**	**6.6**	2.2	**8.4**	1.2	4.6	4	**98**	**27.6**	1	4.2	**14**	1	**49**	4.5	**11**	**7.4**	**10**	3.2	**36.1**	0.9	**68.8**	**55.9**	**77**	**25.4**	3.5	1.1	2	5.9	2.6	6.3	**170**	**11.5**	**7.9**	3.9	**30**	**13**
AST, U/L	^c^	23	35		24	29	24	30	55	**58**	38	77	30	**58**	31	**62**	29		33	**60**	24	**50**	28	**59**	**43**			**97**	30	**121**	23		**47**		**54**	**58**	**54**	**58**	36
ALT, U/L	^d^	27	25		29	33	17	**48**	**124**	34	40	**56**	27	**27**	20	27	30	16	17	25	14	**48**	18	35	34		25	**50**	34	**74**	20		26		35	37	16	25	31
Gamma-GT	^e^		27		**55**		18		52	22	28		30		18		31		19		18	14	7	14	16		23		28	60	17		58		31		**45**		
Erytrocytes																																							
C16:0 DMA	6.8–11.9 %	7.9	**6.2**		6.8	8	6.9	**4.9**	**5.7**	8.2	9.2	**1.5**	7.3	7.3	7.6	8.4	**6.3**	7.6	7.5	9.6	**6.4**	7.5	**6.5**	7.7	7.2	9.4	7.9				8.7	**6.7**	**4.3**	8.3	**6.5**	**6.6**	**5.2**	8	7.3
C18:0 DMA	10.6–24.9 %	16	14.7		18.8	16	15.8	12	12.2	18	22.4	**7.9**	19.6	17	15.5	21	15.4	17.9	17.7	18.3	17.1	20	16.3	17.9	17.1	21.2	19.9				17	16.8	**9.3**	18	15.3	11.3	**10.1**	17	19.4
Urine ^f^																																							
Oxalic acid	0.02–0.06		**0.06**				**0.06**				0.05		0.02		0.04		0.01	0.11	0.01	**0.16**	0.02	**0.1**	0.04	**0.09**	0.03		0.06						**0.08**				**0.15**	**0.1**	0.04
Glycolic acid	20–140		115				120				139		60		124		58	199	102	34	**261**	**209**	118	**266**	**356**		70						126					**286**	**231**

*Abbreviation: AST* aspartate aminotransferase, *ALT* alanine aminotransferase, *CA* cholic acid, *CDCA* chenodeoxycholic acid, *DHCA* dihydroxycholestanoic acid, *DMA* dimethyl acetal, *THCA* trihydroxycholestanoic acid

(A) sibs; (B) sibs; (C) sibs

All the results outside the reference range are depicted in bold

^a^Age at first (complete) biochemical analysis

^b^Age at last complete biochemical analysis

^c^♂/♀0–40, 0–1 year: 0–95; 1–3 years: 0–60

^d^♂0–45, ♀0–34; 0–1 year: 0–65

^e^♂0–60, ♀0–40; 1–15 year 0–56

^f^Oxalic acid: mmol/mmol creatinine, glycolic acid: mmol/mol creatinine

**Table 5:** Biochemical analyses in cultured skin fibroblasts from ZSD patients

	Ref. range	No. 1 (A)	No. 2	No. 3(A)	No. 4	No. 5	No. 6	No. 7	No.8 (B)	No. 9 (B)	No. 10	No. 11	No. 12	No.13 (C)	No. 14	No.15	No.16 (C)	No.17	No. 18	No. 19
Fibroblasts																				
C22:0, μmol/g protein	3.84–10.20		**3.02**	**2.76**	5.47	**2.36**	**3.35**	4.67	**3.01**	**3.6**	4.17	5.18	**3.33**		**2.9**		**2.73**	5.45	**2.91**	**3.68**
C24:0, μmol/g protein	7.76–17.66		**7.68**	8.8	11.04	**5.41**	8.74	8.64	13.16	11.16	9.59	14.14	8.46		8.66		**6.12**	12.48	8.37	10.64
C26:0, μmol/g protein	0.18–0.38		**0.76**	**1.25**	0.27	**1.23**	**0.98**	**0.56**	**3.08**	**2.52**	**0.96**	**1.45**	**0.4**		**1.35**		**0.77**	**1.69**	**1.72**	**1.73**
C26:0/C22:0, ratio	0.03–0.07		**0.25**	**0.45**	0.05	**0.52**	**0.29**	**0.12**	**1.02**	**0.7**	**0.23**	**0.28**	**0.12**		**0.47**		**0.28**	**0.31**	**0.59**	**0.47**
C26:0β-oxidation, pmol/(h*mg protein)	1214–1508			**424**	2069	**213**	**482**	**836**	**581**	**722**	**489**	**399**	**1118**		**714**		**322**	**671**	**3**	**317**
Pristanic acid β-oxidation, pmol/(h*mg protein)	675–1121			**9**	736	**57**	**27**	**107**	**171**	**131**	**37**	**61**	**430**		**178**		**32**	**118**	**1**	**17**
Phytanic acid α-oxidation, pmol/(h*mg protein)	44–82				**40**	**6**		**4**	**13**	**8**	**11**	**7**	**31**				**3**	**8**		**1**
DHAPAT activity, nmol/(2 h*mg protein)	5.8–12.3		**1.2**	**3.5**	12	**1.5**	**2**	**4.3**	**4.8**	**2.8**	**3.3**	6.2	8.4	**1.72**	**2.9**	**0.7**	**2**	**1.6**	**2**	**2.5**
Catalase immunofluorescence	punctate	mosaic	mosaic		punctate	enlarged		mosaic	enlarged	enlarged	enlarged	diffuse	mosaic				diffuse	diffuse	enlarged	diffuse

*Abbreviation: DHAPAT* dihydroxyacetonephosphate-acyltransferase, *diffuse* cytoplasmic catalase distribution, *punctuate* peroxisomal catalase distribution, *mosaic* both fibroblasts with diffuse and punctuate distribution, *enlarged* less peroxisomes and enlarged in size

Control fibroblasts show a punctuate catalase distribution, as catalase is imported into functional peroxisomes

(A) sibs; (B) sibs; (C) sibs

All the results outside the reference range are depicted in bold

In general, patients had reduced levels of fat soluble vitamins (A, D, E and K) and a coagulopathy, due to a combined vitamin K malabsorption and liver dysfunction. The majority of patients had a low-phytanic acid and low-fat diet and 14 patients received vitamin A, D, E and K supplementation.

## Discussion

The ZSDs have long been considered lethal in infancy or early childhood, based on the original description of Zellweger syndrome (Bowen et al 1964). In this retrospective study, we describe a large cohort of patients with a ZSD and show that the natural history is highly variable with a distinct subgroup surviving well into adulthood. The phenotypic spectrum is therefore much wider, with implications for counseling of patients and their families.

We attempted to identify clinical, biochemical, genetic and/or MRI characteristics typical for this subgroup with long survival. We divided the patients in this cohort into two categories based on the degree of communication. Patients in group 1 (patients 1–12) were able to communicate with structured grammatical speech and patients in group 2 (patients 13–19) did not achieve structured speech. The correlation between the phenotype and genotype, at least with respect to the mild *PEX1* c.2528G>A and *PEX26* c.292C>T mutation in its homozygous form versus the more severe compound heterozygosity (*PEX1* c.2097insT+*PEX1* c.2528G > A), which was described previously (Rosewich et al 2005) (Bader et al 2000), was also seen in our study.

In previous case reports, age at first symptoms varied from 3 to 12 years and age at diagnosis varied from 10 to 51 years (Rosewich et al 2005) (Mignarri et al 2012) (Matsui et al 2013) (Raas-Rothschild et al 2002) (Régal et al 2010). The average age at diagnosis in our cohort was at a much earlier age of 5.2 years. Hence, we had the possibility to monitor the clinical and biochemical spectrum over a relatively long period of time.

Hearing impairment, an important characteristic of ZSD patients (Moser et al 1995), was not reported in some of the mild adult patients described in previous reports (Régal et al 2010) (Sevin et al 2011) (Steinberg et al 2009), but all patients in our cohort had impaired hearing and vision. The predominant neurological symptom in the adult patients is a gait disorder, caused by combinations of a cerebellar syndrome, pyramidal tract dysfunction and peripheral neuropathy. Unexpectedly, we observed a high prevalence of signs of peripheral neuropathy in adolescence/adulthood (11/19), especially in group 2 (6/7), while none of these patients presented these symptoms in childhood. The majority of patients was diagnosed with a peripheral neuropathy on clinical grounds. Confirmatory nerve conduction studies, however, were only performed in two patients, which is a limitation of our study. As reported previously (England et al 2005), the combination of signs and symptoms have a relatively good accuracy for diagnosing a peripheral neuropathy. It is noteworthy that two patients developed a demyelinating peripheral neuropathy. This contrasts with another peroxisomal disease, X-linked adrenoleukodystrophy, in which axonal rather than a demyelinating peripheral neuropathy is usually present (van Geel et al 1996) (Engelen et al 2011) (Chaudhry et al 1996).

Previous MRI studies showed progressive cerebral demyelination in peroxisome biogenesis disorder patients with a mild phenotype, mostly in the cerebellum, brainstem, posterior limb of the internal capsule and posterior cerebral white matter (Barth et al 2004). In this study, the major MRI abnormalities (i.e., white matter hyperintensities) were found in the central white matter of both cerebellar hemispheres and/or areas surrounding the dentate nuclei on T2-weighted images (9/16). These lesions can also be found in patients with D-bifunctional protein deficiency, an isolated peroxisomal β-oxidation defect, with prolonged survival (i.e., >7.5 years) (Ferdinandusse et al 2006). Normal MRI was found in five patients. Symptomatic leukoencephalopathy was present in seven patients (2, 5–6, 9, 12, 17–18) and silent leukoencephalopathy in two (3, 8). Overall, individuals in both groups presented similar findings on MRI ranging from normal findings to leukoencephalopathy.

Liver dysfunction is a common feature in ZSD patients. However, in this cohort only four patients (in group 2) had liver cirrhosis/fibrosis. The prevalence of hyperoxaluria (2/14) and hyperglycolic aciduria (3/13) was much lower than previously reported in ZSD patients older than 1 year (van Woerden et al 2006). Only patient 18 suffered from nephro- and urolithiasis.

In our cohort 12 patients were clinically stable in recent years and seven showed a progressive disease course. The disease progression becomes apparent in adolescence (age 12–16 years), with gait disturbance being the most prominent symptom (in 7/7).

Seventeen of the 19 patients had a typical ZSD biochemical phenotype in blood at the time at diagnosis. At latest follow-up we observed normal blood levels of several peroxisomal biomarkers in patients (Table 4). Normal levels of some parameters have already been reported by others (Sevin et al 2011) (Ebberink et al 2010). Importantly, our study is the first to show a decline in the levels of these metabolites with age and in some patients even a complete normalization. In particular the levels of DHCA, THCA, and pipecolic acid were found to decline during life and they eventually normalized. In some patients we noted a decrease in these parameters as well as in liver enzymes. This suggests that improved liver function might play a role in the decrease of these metabolites, as they are predominantly synthesized in the liver (Ferdinandusse and Houten 2006). However, there were also patients with normal liver function and elevated levels of abnormal peroxisomal metabolites in childhood. In these patients we also observed a decline in these peroxisomal parameters, meaning that this decline cannot be entirely attributed to normalization of liver functions. The decline in DHCA and THCA levels may also be caused by a decreased synthesis of bile acids with increasing age (Einarsson et al 1985). Furthermore, we observed strong fluctuation of several parameters in the majority of the patients. The concentration of peroxisomal metabolites can fluctuate between normal and abnormal (Fig. 1). Overall, the diagnosis ZSD would be missed in two patients (2 and 11) at last follow-up and in patient 4 at first analysis based on the C26:0, bile acid levels, pristanic- and phytanic acid concentrations in plasma.

Our data suggest that a ZSD cannot be excluded by biochemical testing in plasma alone, and that in some individuals (i.e., patient 2, 4, and 11) a complete analysis in skin fibroblasts (including culturing fibroblasts at 40 °C (Ebberink et al 2012)) is indicated if clinical suspicion is high. Skin fibroblast examination is also necessary to discriminate between a ZSD or a single-enzyme deficiency, which is diagnosed in at least 15 % of the individuals presenting with a ZSD clinical phenotype (Steinberg et al 2003). Recently, D-bifunctional protein-deficiency has been identified by whole-exome sequencing in seven adults without detectable biochemical abnormalities in blood (Pierce et al 2010) (McMillan et al 2012) (Lines et al 2014). Because of considerable overlap between Usher syndrome and milder ZSD phenotypes, i.e., the combination of deafness and retinitis pigmentosa, individuals suspected to have Usher syndrome should be screened for peroxisomal dysfunction (Raas-Rothschild et al 2002).

Efforts to correlate biochemical, cellular, and molecular characteristics of ZSD patients to their clinical outcome, mainly in terms of survival, have provided valuable information. Gootjes et al suggested that DHAPAT and C26:0 β-oxidation activity are predictors of survival in patients with a ZSD. However, these measurements in cultured skin fibroblasts could only be used to roughly predict survival of <1 year or >5 years (Gootjes et al 2002). Despite these associations, prediction of prognosis remains challenging and is influenced by many other variables (e.g., quality of supportive care). We evaluated how the parameters of peroxisomal dysfunction relate to disease severity and long-term progression. We noticed higher levels of abnormal peroxisomal metabolites (e.g., DHCA, THCA, and C26:0) in group 2 during childhood, but there were no differences in adulthood. Concentrations of pipecolic acid were higher in those patients with a progressive disease course. Patients in group 2 had higher levels of C26:0 in plasma than patients in group 1 throughout life (Fig. 1d). Ferdinandusse et al reported that C27-bile acid intermediates (DHCA/THCA) induce apoptosis, decrease cell viability and are more toxic than C24-bile acids (Ferdinandusse et al 2009). It is noteworthy that the most severely affected patient (18) had extremely high plasma levels of DHCA and THCA ranging from 50 to 130 μmol/l in the first year of life. The patients with a less severe phenotype presented with DHCA and THCA levels of approximately 10 and 2 μmol/l, respectively. However, on the basis of the present study, we were unable to identify a correlation between the concentration of abnormal peroxisomal metabolites, skin fibroblast data and the severity of the clinical phenotype or progression of the disease. The metabolites measured in plasma probably do not reflect the level of accumulation in organs. This suggests that the wide spectrum of clinical presentations may be related to varying amounts of residual peroxisomal enzymatic activities in target tissue. Further studies are therefore required to determine the precise role of the peroxisomal biomarkers in the pathogenesis of ZSDs. The recently constructed PEX1 mouse model (Hiebler et al 2014) may be a valid model to study these relations.

In summary, we describe the natural history of a cohort of ZSD patients who reached adulthood and represent a distinct subgroup within the ZSDs. A high percentage of patients show pyramidal symptoms with or without peripheral neuropathy. Some patients with prolonged survival present an insidiously progressive disease course, despite normalization of biomarkers for peroxisomal disease measured in plasma and erythrocytes. This study emphasizes that ZSDs should no longer be considered solely as a paediatric disease, but rather as a slowly progressive disease with patients surviving into adulthood. This study is important for the interpretation of future therapeutic trials and for those involved in the clinical care of adult patients.

## Abbreviations

DHAPAT: dihydroxyacetonephosphate-acyltransferase
DHCA: dihydroxycholestanoic acid
DMA: dimethyl acetal
THCA: trihydroxycholestanoic acid
VLCFAs: very long-chain fatty acids
ZSDs: Zellweger spectrum disorders
